# Practical problems of measuring depth of submucosal invasion in T1 colorectal carcinomas

**DOI:** 10.1007/s00384-015-2403-7

**Published:** 2015-10-02

**Authors:** Yuta Kouyama, Shin-ei Kudo, Hideyuki Miyachi, Katsuro Ichimasa, Tomokazu Hisayuki, Hiromasa Oikawa, Shingo Matsudaira, Yui J. Kimura, Masashi Misawa, Yuichi Mori, Kenta Kodama, Toyoki Kudo, Takemasa Hayashi, Kunihiko Wakamura, Atsushi Katagiri, Eiji Hidaka, Fumio Ishida, Shigeharu Hamatani

**Affiliations:** Digestive Disease Center, Showa University Northern Yokohama Hospital, 35-1 Chigasaki Chuo, Tsuzuki-ku, Yokohama City, Kanagawa 224-8503 Japan; Department of Pathology, The Jikei University School of Medicine, Tokyo, Japan

**Keywords:** Risk factor, T1 colorectal carcinoma, Lymph node metastasis, Invasion depth

## Abstract

**Purpose:**

Submucosal invasion depth (SID) in colorectal carcinoma (CRC) is an important factor in estimating risk of lymph node metastasis, but can be difficult to measure, leading to inadequate or over-extensive treatment. Here, we aimed to clarify the practical aspects of measuring SID in T1 CRC.

**Methods:**

We investigated 568 T1 CRCs that were resected surgically at our hospital from April 2001 to December 2013, and relationships between SID and clinicopathological factors, including the means of measurement, lesion morphology, and lymph node metastasis.

**Results:**

Of these 568 lesions, the SID was ≥1000 μm in 508 lesions. SIDs for lesions measured from the surface layer were all ≥1000 μm. Although lesions with SIDs ≥1000 μm were associated with significantly higher levels of unfavorable histologic types and lymphovascular infiltration than shallower lesions, a depth of ≥1000 μm was not a significant risk factor for lymph node metastasis (LNM) (6.7 vs. 9.8 %; *P* = 0.64), and no lesions for which the sole pathological factor was SID ≥1000 μm had lymph node metastasis. Protruded lesions showed deeper SIDs than other types.

**Conclusions:**

Although we found several problems of measuring SID in this study, we also found, surprisingly, that SID is not a risk factor for lymph node metastasis, and its measurement is not needed to estimate the risk of lymph node metastasis.

## Introduction

In T1 colorectal cancer (CRC), deep submucosal invasion thought to signify malignant potential and submucosal invasion depth (SID) are considered important predictors of risk of lymph node metastasis (LNM) [1–9]. Haggitt’s classification criteria defined level 2 as a risk of LNM [10–12]. Kudo’s Classification of Degree of Submucosal Invasion defined sm1c, sm2, and sm3 as risk factors for LNM [13, 14]. Although the European Society for Medical Oncology (ESMO) guidelines recommend surgical resection after endoscopic resection if deeper infiltration into the submucosa is found [15], deeper infiltration into the submucosa is not well defined in these guidelines. Kitajima et al. established the method for measuring SID and reported no LNM were found in lesions with SID <1000 μm [5]. Therefore, SID has been thought to be an important factor in estimating LNM risk. The clinical guidelines of the Japanese Society for Cancer of the Colon and Rectum (JSCCR) describe the method of measuring an SID and advise additional surgical colectomy with lymph node dissection after endoscopic treatment for lesions with SID ≥1000 μm [16]. This “1000-μm rule” is also recommended as a criterion for additional colectomy in many previous papers [8, 17, 18] and is used as a gold-standard indicator for endoscopic diagnoses, such as magnifying endoscopy or image-enhanced endoscopy [19–23].

Overuse of surgery has been recently addressed as a major problem in T1 CRCs [24, 25]. As the percentage of T1 CRCs that lead to LNM is estimated as 6.3–17 % [1–7, 26, 27], many patients who undergo additional colectomies do not have LNM and might not need further resection. Moreover, several studies report that among patients with SID ≥1000 μm but no risk factors for LNMs (lymphovascular infiltration, unfavorable histological types, or tumor budding), few develop LNM [5, 27–29]. As endoscopic techniques progress, endoscopic treatment is becoming more widely acceptable for lesions with SID ≥1000 μm [17, 30, 31]. Problems in measuring SID are also reported in some studies, and both measuring methods and lesion morphology could affect measurement results [32, 33]. However, as these published cases and reports are few, we performed this larger-scale study to clarify the practical issues and significance of measuring SID in T1 CRCs.

## Methods

### Subjects

**Fig. 1:**
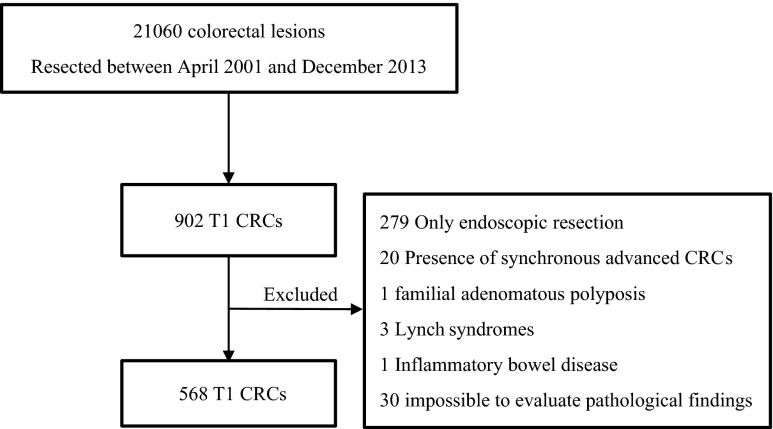
Patient flow chart. *CRC* colorectal carcinoma

**Fig. 2:**
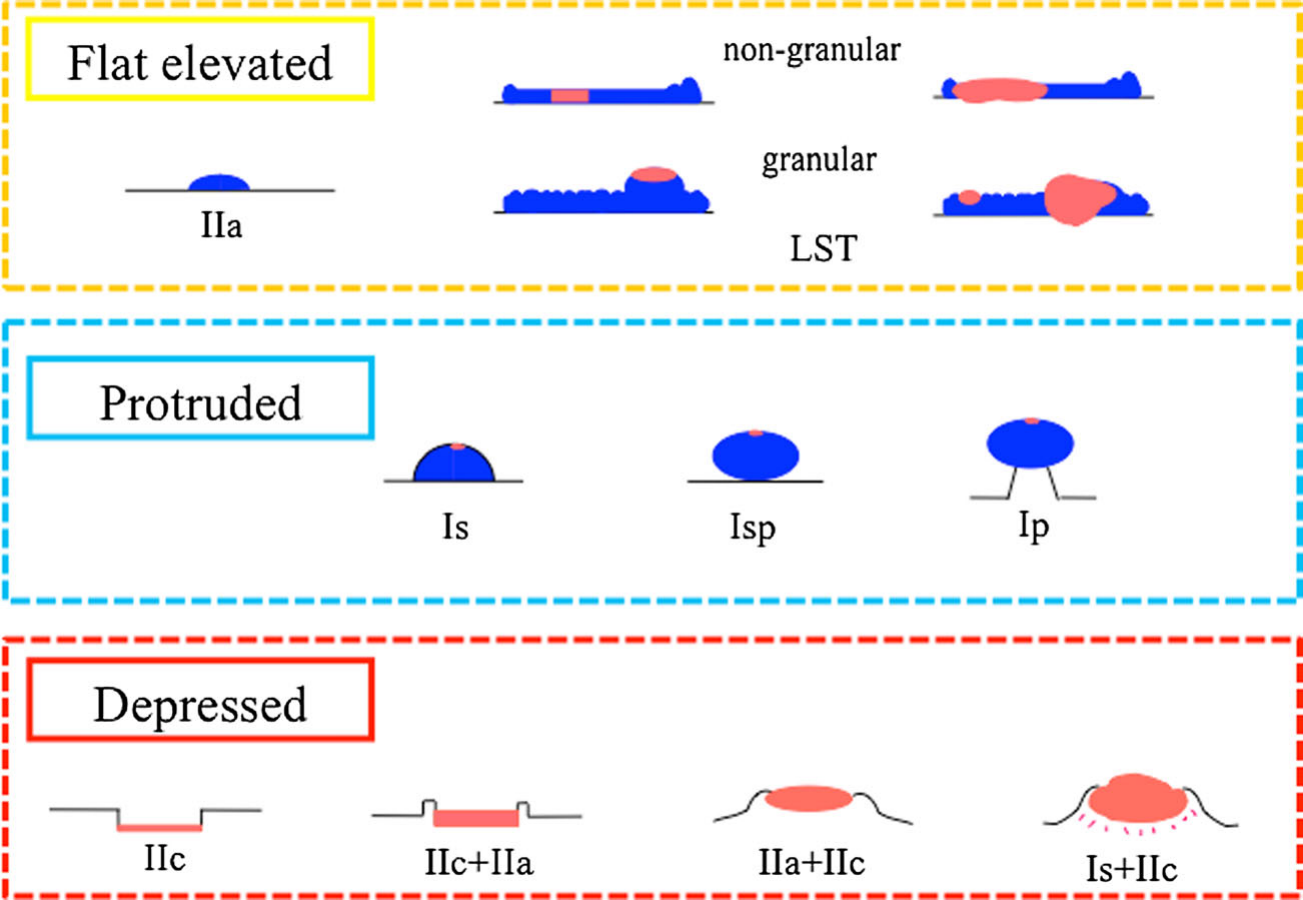
Kudo’s morphological/development classification

In the present study, we analyzed T1 CRCs that were resected at Showa University Northern Yokohama Hospital in Japan from April 2001 to December 2013. A total of 21,060 colorectal neoplasms, excluding advanced carcinomas, had been resected endoscopically or surgically. Of these, 902 were T1 CRCs. To evaluate precise pathological diagnoses and LNM outcomes, we excluded lesions from 334 patients, including 279 patients who underwent only endoscopic resection, 20 who had synchronous advanced CRCs, one with familial adenomatous polyposis, three with Lynch syndromes, one with inflammatory bowel disease, and 30 whose specimens were impossible to evaluate pathologically in detail because of damage or loss (Fig. 1). In total, 568 patients with T1 CRC were included in this study, of whom 295 underwent surgical resection and lymph node dissection as first-line treatment, and 273 underwent first-line endoscopic resection followed by additional surgery with lymph node dissection. None of the patients received preoperative radiotherapy or neoadjuvant chemotherapy. We found each patient’s age, sex, and tumor location from their hospital records. For the morphological classification, we divided the lesions into three types: flat elevated type (0–IIa or laterally spreading tumor [LST]) [13]; protruded type (0–Is, Isp, or Ip); and depressed type (0–IIc, IIc + IIa, IIa + IIc, Is + IIc, or Ip + IIc); Fig. 2 [34, 35].

### Histologic examination

All resected specimens were immediately fixed in 10 % buffered formalin solution, observed with a focus on pit pattern using a stereomicroscope, and dissected at the point where the deepest invasion area could be exposed on the cut end surface. The other histological specimens were subsequently dissected into parallel 2- to 3-mm-thick sections and stained with hematoxylin and eosin (H&E). An experienced pathologist (S.H.) examined all the specimens histologically. Histologic grade was diagnosed based on the World Health Organization Classification of Tumors [36] and the current JSCCR guidelines. Unfavorable histological types were poorly differentiated adenocarcinoma (Por) or mucinous carcinoma (Muc) at any part of the lesion; favorable histological types were well or moderately differentiated adenocarcinoma. Double staining with H&E and Victoria blue (Muto Pure Chemicals Co., Ltd., Tokyo, Japan) was used to diagnose vascular infiltration. Lymphatic infiltration and status of muscularis mucosae were evaluated using H&E staining and immunostaining with D2-40 antibody (Dako North America Inc., Carpinteria, CA, USA) and desmin antibody (Dako North America Inc.), respectively. Tumor budding was defined as a cancer cell nest consisting of 1 or ˂5 cells that had infiltrated the interstitium at the invasive margin of the cancer. After selecting the field where budding was the most intensive, buddings were counted in a 0.785-mm^2^ field as observed through an objective lens. Depending on the number of buddings, budding grade was scored as Grade 1: 0–4; Grade 2: 5–9; and Grade 3: ≥10 [8, 16, 37]. Grades 2–3 were defined as tumor budding positive.

**Fig. 3:**
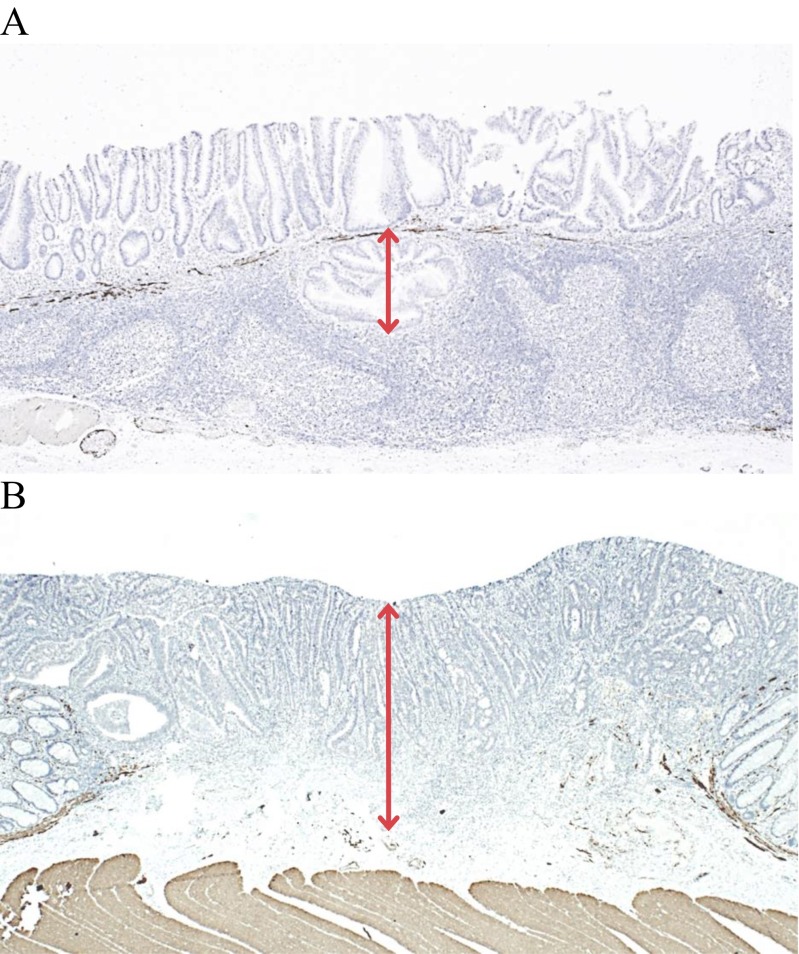
Method used to measure submucosal invasion depth. **a** When identifying or estimating location of the muscularis mucosae, the submucosal invasion depth is measured directly from the line of the muscularis mucosae. **b** When the muscularis mucosae have not been identified, the submucosal invasion depth is measured from the lesion’s surface layer

We measured SID according to the clinical guidelines of the JSCCR [5, 16]. SID can be measured in two ways (Fig. 3). When it was possible to identify or estimate the location of the muscularis mucosae, we measured the SID directly from the line of the muscularis mucosae (method A; Fig. 3a). When the line of the muscularis mucosae was not easily identified because of carcinoma invasion, we measured the SID from the surface layer of the lesion (method B; Fig. 3b).

The lesions were divided into two groups: those with SID <1000 μm and those with SID ≥1000 μm. Between the two groups, we compared incidences of clinicopathological factors: age, sex, tumor location, tumor morphology, tumor size, method of measurement, mean SID, histologic type, lymphatic infiltration, vascular infiltration, tumor budding, and LNM. Mean SID and the LNM rates were calculated for each morphological type. We also assessed correlations between histologic type, lymphatic infiltration, vascular infiltration, tumor budding, and LNM, and SID by depth (0–999 μm, 1000–1999 μm, 2000–2999 μm, 3000–3999 μm, 4000–4999 μm, 5000–5999 μm, 6000–6999 μm, 7000–7999 μm, and ≧8000 μm,). Finally, in lesions with SID ≥1000 μm, we examined correlations between LNM and histologic type, lymphatic infiltration, vascular infiltration, and tumor budding.

### Statistical analysis

Data are presented as the mean ± standard deviation. Differences were analyzed using the *χ*^2^ test, Fisher’s exact test, Student’s *t* test, or Ryan multiple comparison as necessary. Statistical significance was defined as a *P* < 0.05. All statistical analyses were performed using R version 2.10.0. (http://www.r-project.org).

The ethics committee of our hospital approved the study protocol (approval number; 1408–01). Informed consent was obtained from all patients before endoscopy or surgery. This study was registered in the University Hospital Medical Network Clinical Trials Registry (UMIN R000017608). A summary of this study was presented at the United European Gastroenterology Week, Vienna, Austria on October 21, 2014 (oral presentation).

## Results

### Clinicopathological characteristics

Initial or additional surgery with nodal dissection was performed for the 568 patients, who included 352 (62.0 %) men and 216 (38.0 %) women with a mean age of 65.0 ± 11.3 years (range 31–93 years). Mean tumor size was 20.6 ± 11.4 mm (range 4–100 mm). Tumors included 151 flat elevated type, 250 protruded type and 167 depressed type lesions, with 401 lesions located in colon and 167 in rectum, and 54 (9.4 %) lesions associated with LNM. Unfavorable histologic types were seen in 106 (18.7 %) lesions, lymphatic infiltration was positive in 237 (41.7 %) lesions, vascular infiltration was positive in 189 (33.5 %) lesions, and tumor budding was positive in 158 (27.8 %) lesions.

We found 60 lesions (10.6 %) with SID <1000 μm and 508 (89.4 %) with SID ≥1000 μm. SID was measured from the muscularis mucosa in 168 (32.2 %) lesions (method A) and from the surface in 385 (67.8 %) lesions (method B). Among the lesions measured using method A, 60 had SIDs <1000 μm and 123 had SIDs ≥1000 μm. All lesions measured using method B invaded deeper than 1000 μm.

### SID and clinicopathological factors

The SID <1000 μm group had a lower rate of rectal T1 carcinomas than did the SID ≥1000 μm group (15.0 vs. 31.1 %; *P* = 0.010), depressed type lesions (13.3 vs. 31.3 %; *P* = 0.004), unfavorable histological types (8.3 vs. 19.9 %; *P* = 0.034), lymphatic infiltration (28.3 vs. 43.3 %; *P* = 0.027), and vascular infiltration (10.0 vs. 36.0 %; *P* < 0.001; Table 1). The two groups did not significantly differ by age, sex, or tumor size. The two groups also did not significantly differ by rate of LNM (6.7 vs. 9.8 %; *P* = 0.64) or tumor budding (20.0 vs. 28.7 %; *P* = 0.17).

**Table 1:** Submucosal depth (<1000 or ≥1000 μm) and clinicopathological factors

	<1000 μm (*n* = 60)	≥1000 μm (*n* = 508)	*P* value
Age (years), mean (SD)	65.5 ± 12.6	64.9 ± 11.2	0.7099
Male sex	42 (70.0)	310 (61.0)	0.2062
Location (rectum)	9 (15.0)	158 (31.1)	0.0103
Morphology (depressed type)	8 (13.3)	159 (31.3)	0.0040
Tumor size (mm), mean (SD)	19.5 ± 11.9	20.7 ± 11.3	0.4325
Method of measurement (A)	60 (100)	123 (24.2)	<0.001
SID (μm), mean (SD)	406 ± 302	3680 ± 2233	<0.001
Histologic type (por or muc)	5 (8.3)	101 (19.9)	0.0341
Lymphatic infiltration (+)	17 (28.3)	220 (43.3)	0.0271
Vascular infiltration (+)	6 (10.0)	183 (36.0)	<0.001
Budding (+)	12 (20.0)	146 (28.7)	0.1722
Lymph node metastasis (+)	4 (6.7)	50 (9.8)	0.6401

Data are expressed as the number of patients (%) unless otherwise indicated

*Por or muc* poorly differentiated adenocarcinoma or mucinous carcinoma, *SD* standard deviation, *SID* submucosal invasion depth, *Budding* tumor budding

**Fig. 4:**
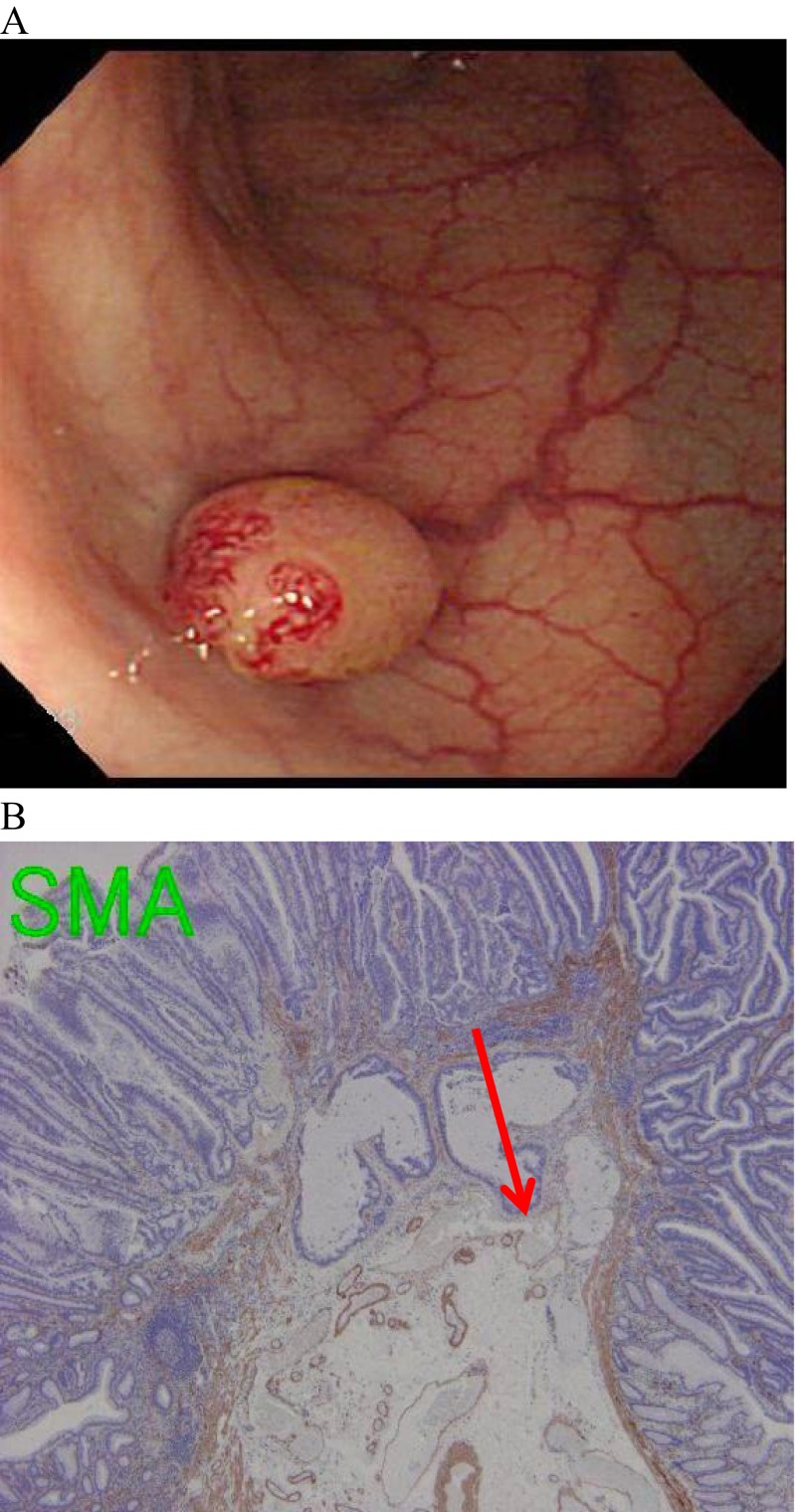
A case of lymph node metastasis with the submucosal invasion depth <1000 μm. **a** A 7-mm sessile lesion was detected in the sigmoid colon. **b** The submucosal invasion depth was 800 μm (the *arrow*)

Figure 4 shows a lesion that metastasized to a lymph node even though its SID was <1000 μm. It was found in a 50-year-old male patient as a sessile lesion, 7 mm in diameter, in the sigmoid colon. Its SID was 800 μm, with positive lymphatic infiltration, and negative vascular infiltration and budding. This patient was treated with an additional surgical colectomy.

### SID and morphology

Mean SIDs of flat elevated type lesions, protruded type lesions, and depressed type lesions were 2393 ± 1652 μm, 4089 ± 2796 μm, and 2974 ± 1503 μm, respectively (Table 2). The mean SID of protruded lesions was greater than that of other types (*P* < 0.01). Rates of LNM for flat elevated (7.3 %), protruded (11.2 %), and depressed (9.0 %) lesions did not significantly differ (*P* = 0.42).

**Table 2:**
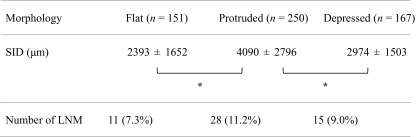
Mean depth of invasion and incidences of lymph node metastasis by morphology of the lesion

*LNM* lymph node metastasis, *SID* submucosal invasion depth

*P < 0.05

### Actual measured SID value and pathological factors

Data on rates of unfavorable histologic type, lymphatic infiltration, vascular infiltration, tumor budding, and LNM according to SID are shown in Table 3. Rates of unfavorable histologic type, lymphatic infiltration, or budding did not significantly differ by SID. The rate of vascular infiltration was significantly less in lesions with a SID 0–999 μm than in lesions with a SID of 3000–3999 (*P* = 0.0002), 4000–4999 (*P* = 0.0008), and 6000–6999 μm (*P* < 0.0001). The rate of LNM was higher in lesions with a SID of 6000–6999 μm than in lesions with a SID of 2000–2999 (*P* = 0.0011).

**Table 3:**
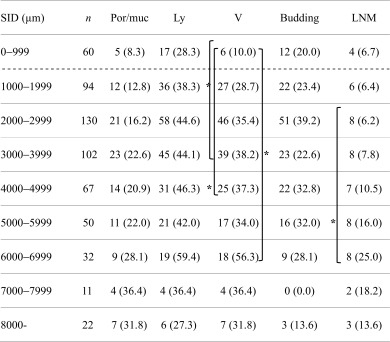
Actual measured depth of submucosal invasion and pathological factors SX

Data are expressed as the number of patients (%)

*SID* submucosal invasion depth, *n* number of lesions, *Por/muc* poorly differentiated adenocarcinoma or mucinous carcinoma, *Ly* lymphatic infiltration, *V* vascular infiltration, *Budding* tumor budding, *LNM* lymph node metastasis

*P < 0.05

### Pathological factors and LNM in the SID ≥1000 μm group

Table 4 shows correlations between LNM and histologic type, lymphatic infiltration, vascular infiltration, and tumor budding in the SID ≥1000 μm group. No patients in this group developed LNM except those who had a pathological factor associated with LNM (i.e., other than LNM). Among patients in this group who had LNM-associated pathological factors, the rate of LNM was 14.5 % (50/345).

**Table 4:** Correlation between lymph node metastasis and pathological factors in lesions with submucosal depth of ≥1000 μm

	Lymph node metastasis	
	+	−
Other pathological factors^a^	50	295
None	0	163

^a^Other pathological factors include lymphovascular permeation, unfavorable histologic types (poorly differentiated adenocarcinoma, signet-ring cell carcinoma, or mucinous carcinoma), or Grade 2–3 budding at the site of deepest invasion

## Discussion

In the present study, we addressed some practical aspects of measuring SID and clarified the significance of SID as a risk factor for LNM.

**Fig. 5:**
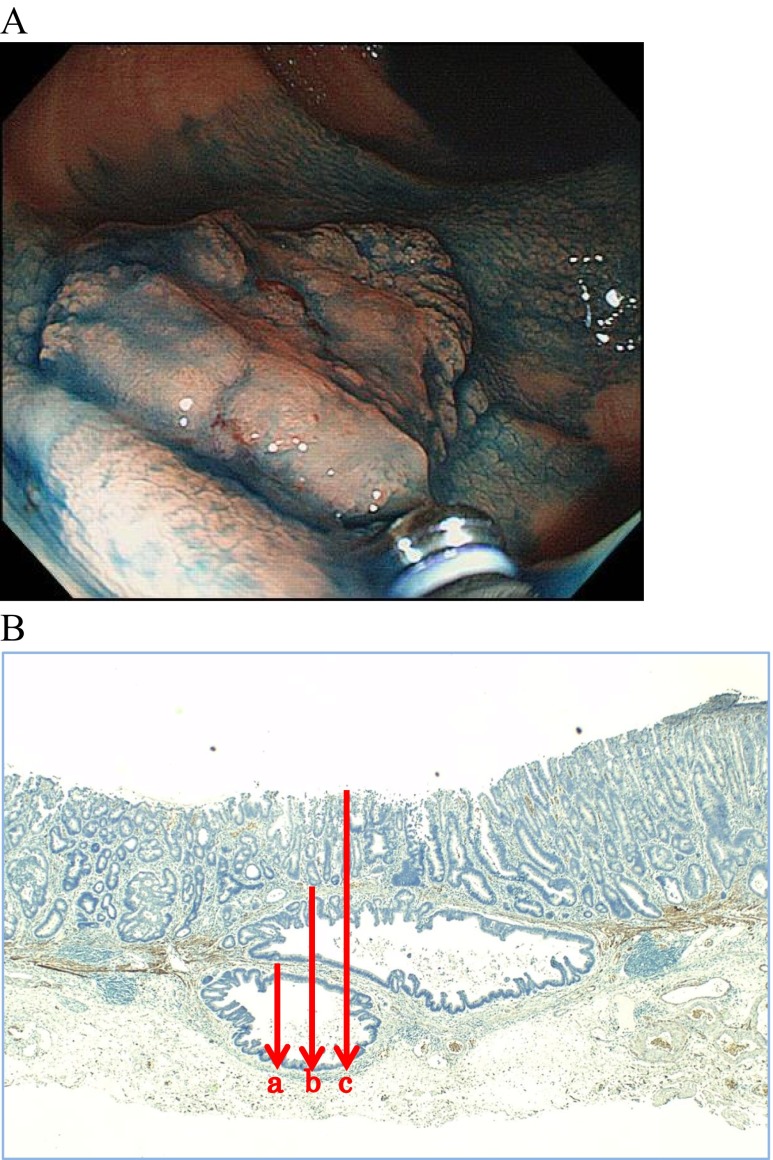
A case in which determining the submucosal invasion depth measurement baseline was difficult. **a** A 23-mm non-granular LST located in the ascending colon. **b** The pathological finding of desmin antibody staining. The submucosal invasion depth was 630 μm when measured from the *lower line* (Fig. 5b, *a*), 1325 μm when measured from the *upper line* (Fig. 5b, *b*), and 2150 μm when measured from the *surface* of the lesion (Fig. 5b, *c*)

First, the method of measuring SID affects the results. In the present study, when measuring a SID from the surface layer (method B), the depth was always ≥1000 μm. Therefore, if a pathologist found that the muscularis mucosa of the lesion could not be easily identified, the 1000 μm rule implies that a colectomy with lymph node dissection should be considered without measuring the SID. Yoshida et al. also reported that SIDs measured from the surface layer were deeper than 1000 μm for all lesions [33]. Second, determining the baseline is difficult in some lesions. The muscularis mucosae sometimes show separations, and several baselines can be estimated in some cases. Figure 5, for example, shows a 23-mm non-granular LST located in the ascending colon. As the muscularis mucosae had split up, it was unclear which line we should measure from. The SID was 630 μm when measured from the lower line (Fig. 5a), 1325 μm when measured from the upper line (Fig. 5b), and 2150 μm when measured from the surface of the lesion (Fig. 5c). Whether additional surgical resection is necessary depends on which baseline the SID is measured from—in other words, the assessment of the pathologist who diagnoses the lesion [38].

Third, SID is affected by lesion morphology [39]. SIDs are greater for protruded lesions than for the other two types. Reportedly, flat elevated type lesions, especially LSTs, grow laterally, protruded types often grow upward, and depressed type lesions tend to invade downwards [40]. These growing patterns can affect SID measurements. Therefore, setting the cutoff value at 1000 μm should consider morphology.

**Fig. 6:**
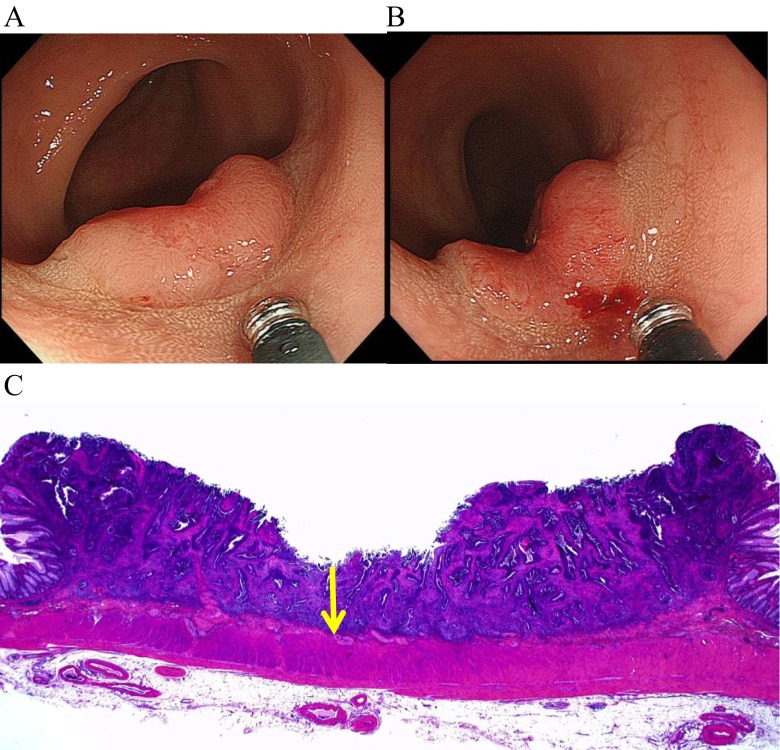
A typical case in which the submucosal invasion depth might become shorter during the carcinoma’s progression. **a** A 19-mm IIa + IIc lesion was detected in the rectosigmoid. **b** The observation of the same lesion 1 month later. **c** The pathological finding with hematoxylin and eosin staining of the lesion. The submucosal invasion depth was 1400 μm in the center of the lesion (the *arrow*)

Fourth, some lesions develop depressed areas, and SID can decrease during the lesion’s progression. Generally, lesions with depressions or ulcerations are more advanced or aggressive [41–46]. Figure 6 shows a lesion in which the center area dimpled over a month’s time and the depth could have become shallower (Fig. 6a, b). Although the SID was only 1400 μm in the center of the lesion, the patient had an LNM. Increased SID does not reflect the lesion’s malignancy or progression or likelihood of LNM. Although the 1000-μm rule quantifies SID as a comprehensible value and provides a clear-cut criterion, its measurement can be difficult to perform and interpret.

SID does provide useful information. The presence of unfavorable histological types or lymphovascular infiltration in lesions with SID <1000 μm was significantly lower than in those with SID ≥1000 μm. Thus, the SID <1000 μm threshold can help estimate the risk of unfavorable histological types or lymphovascular infiltration. However, it is not a significant risk factor for LNM. In the present study, four patients with SID <1000 μm had LNM, two of whom had other LNM-associated factors, such as unfavorable histologic type, lymphovascular infiltration, or tumor budding. This concords with other studies that report patients with SID <1000 μm developing LNM [8, 24, 28, 47, 48]. Several studies have reported deep SID as a risk factor for LNM in patients with T1 CRC [1, 2, 4–9], and others did not evaluate LNM^+^ cases with SID <1000 μm [5, 7, 9, 26], but most of these studies, except for a meta-analysis, had fewer T1 CRC cases than in the present study, even though our study was a single-center study. In the Western World, the discrimination of the submucosal layer into sm1, sm2 (<1000 μm), and sm3 (≥1000 μm) is established to estimate the potential risk of lymph node metastasis [49, 50]. But there also exists the redundancy in the classification same as the “1000-μm rule.”

Moreover, of the cases with SID ≥1000 μm but no LNM-associated pathological factors—unfavorable histological type, lymphovascular infiltration, or tumor budding—none had LNM. In contrast, among cases with SID ≥1000 μm and any of these pathological factors, the rate of LNM was 14.5 %. If the SID is omitted from the risk factors of LNM, we could reduce the number of additional surgeries that are performed according to the 1000-μm rule. Other studies have also reported extremely low incidence of LNM regardless of SID among lesions with no LNM-associated pathological factors [5, 24, 27–29]. Such lesions might be treated only with endoscopic resections. Currently, other factors such as histological type, lymphovascular infiltration, and tumor budding are considered more important than a SID ≥1000 μm. In this study, we addressed several practical problems in measuring SID; therefore, more detailed prospective investigations of LNM risk factors will be needed besides SID. Hereafter, without causing incomplete endoscopic resections, more accurate criteria to recommend additional surgery should be established.

Our study is limited by being a single-center retrospective study, which could reflect some selection bias. However, the pathological specimens were examined in the same manner [48], and with a larger sample size, than any other single-institutional reports published so far. Second, patients treated only by endoscopic resection were excluded from this analysis, as such endoscopically removed lesions occasionally disguise a metachronous recurrence in consideration of skip lymphovascular invasion or discontinuous foci of tumor cells [51]. Risk factors for metachronous recurrence or prognosis are the subject of future investigations. Finally, all patients examined in this study were Japanese. Some biological differences might be seen between Japan and the Western World. Further multicenter trials in the Eastern and Western World would be necessary before the “1000 μm border“ is excluded for risk stratification.

In conclusion, the present study showed several problems of measuring SID and demonstrated that SID is not an important risk factor for LNM. If the surface layer was used as the baseline, all cases would be ≥1000 μm. SID is associated with lesion morphology, and it can sometimes become shorter in progression of the lesions. Some lesions with SID <1000 μm had LNM, whereas SID ≥1000 μm was not a significant risk factor for LNM. Moreover, among lesions with SID ≥1000 μm but no pathological factors associated with LNM, none had LNM. SID should be reconsidered as a risk factor for LNM, or an indicator for additional surgery. We need to devise more accurate methods to assess lesions at high risk for LNM without measuring SID.
